# Paraneoplastic immunoglobulin A nephropathy and associated focal segmental glomerulosclerosis in asymptomatic low volume B-cell lymphoma – a case report

**DOI:** 10.1186/s12882-018-1034-y

**Published:** 2018-09-10

**Authors:** Monica Suet Ying Ng, Leo Francis, Elango Pillai, Andrew John Mallett

**Affiliations:** 1 Kidney Health Service, Royal Brisbane and Women’s Hospital, Brisbane, Australia; 2 Faculty of Medicine, The University of Queensland, Brisbane, Australia; 3 Department of Anatomical Pathology, Pathology Queensland, Royal Brisbane and Women’s Hospital, Brisbane, Australia; 4 Haematology and Bone Marrow Transplantation Service, Royal Brisbane and Women’s Hospital, Brisbane, Australia

**Keywords:** Paraneoplastic, Glomerulonephritis, Lymphoma, Immunoglobulin A nephropathy, Proteinuric, Non-Hodgkin

## Abstract

**Background:**

Paraneoplastic glomerulonephritis is rare in haematological malignancies and tends to manifest as minimal change disease, membranous glomerulonephritis or membranoproliferative glomerulonephritis. We present the first report of immunoglobulin A nephropathy and associated focal segmental glomerulosclerosis in a patient with asymptomatic low grade B-cell lymphoma.

**Case presentation:**

A 53 year old gentleman presented with nephrotic range proteinuria (urine protein creatinine ratio of 662 mg/mmol) on a background of type 2 diabetes mellitus (glycosylated haemoglobin: < 6%), hypertension, obesity (body mass index: 47.6 kg/m^2^) and degenerative spine disease. Bone marrow biopsy diagnosed a low grade B-cell lymphoma and renal biopsy was consistent with immunoglobulin A nephropathy. Lymphoma treatment with six cycles of cyclophosphamide/ rituximab/ prednisolone led to normalisation of urinary protein excretion (urine protein creatinine ratio: 14 mg/mmol at 26 months post-chemotherapy).

**Conclusion:**

Paraneoplastic immunoglobulin A nephropathy can occur with a broad range of haematological malignancies regardless of stage. This case illustrates the importance of meticulous haematological system work-up for patients presenting with immunoglobulin A nephropathy. Recognition of paraneoplastic immunoglobulin A nephropathy and early diagnosis of associated malignancy can be life-saving.

## Background

Immunoglobulin A nephropathy (IgAN) is the most common cause of glomerulonephritis worldwide, accounting for up to 30–50% of biopsy proven glomerulonephritis [1]. It is distinguished by IgA-dominant or co-dominant mesangial immune deposits which are visualised using immunohistochemistry [2]. Secondary IgAN has been associated with various autoimmune conditions (systemic lupus erythematosus [3], psoriasis [4], inflammatory bowel disease [5]), infections (brucellosis [6], viral hepatitis [7], tuberculosis [8]) and malignancies (lymphoma, leukaemia, solid organ tumours) [1]. Lymphoma-associated IgAN is rare with the majority of reported cases involving aggressive Hodgkin and T-cell lymphomas [9–11]. Here, we present the first report of IgAN and associated focal segmental glomerulosclerosis (FSGS) in a patient with asymptomatic low grade B-cell lymphoma. This case broadens the types of lymphomas associated with IgAN and demonstrates that lymphoma-associated IgAN can occur even with low volume disease.

## Case presentation

In October 2014, a 53 year old male Caucasian administration officer was referred to a tertiary renal outpatient clinic for assessment of nephrotic range proteinuria. On review, he described occasional ankle oedema over the past year but otherwise felt well. His past medical history was significant for type 2 diabetes mellitus which was treated in 2007 with 15 kg of weight loss (glycosylated haemoglobin < 6% since 2013). He denied symptoms of macrovascular or microvascular complications. The patient also had hypertension (diagnosed > 10 years prior to review), obesity (body mass index: 47.6 g/m^2^) and degenerative spine disease. His medications were irbesartan 300 mg once daily, hydrochlorothiazide 25 mg once daily, tramadol 50 mg three times a day and meloxicam 7.5 mg as needed. His family history was significant for type 1 diabetes mellitus in his father and paternal uncle. On examination, his blood pressure was 140/80 mmHg and heart sounds were dual with no murmurs. There was bipedal oedema up to the bottom third of his shins, his jugular venous pressure was not elevated and his chest was clear to auscultation.

Initial laboratory investigation demonstrated haemoglobin 149 g/L, white cell count 7.20 × 10^9^/L, platelets 235 × 10^9^/L, blood urea nitrogen 6.0 mmol/L, serum creatinine 70 μmol/L and serum albumin 32 g/L. Serum electrolytes, liver enzymes, calcium and phosphate were within normal range. Repeated urinalysis did not demonstrate any haematuria, leukocyturia or casts. Urine protein creatinine ratio was 662 mg/mmol (normal in August, 2012, Fig. 1). Serum levels of antinuclear antibody, antineutrophil cytoplasmic antibody, immunoglobulin (Ig) A, cryoglobulins, complement factor C3 and C4 were normal. Human immunodeficiency virus screen, Hepatitis B antigen and hepatitis C antibodies were negative. Serum κ free light chains (FLC) was 18 mg/L (7–22), serum λ FLC was 60 mg/L (8–27) and κ/λ ratio was 0.30 (0.31–1.56). Repeated serum electrophoreses over three months showed persistent κ and λ IgG bands in trace amounts consistent with an inflammatory or reactive pattern. The repeated abnormal serum electrophoreses in the absence of inflammation (normal erythrocyte sedimentation rate and C-reactive protein levels) prompted referral to haematology for further assessment.

**Fig. 1:**
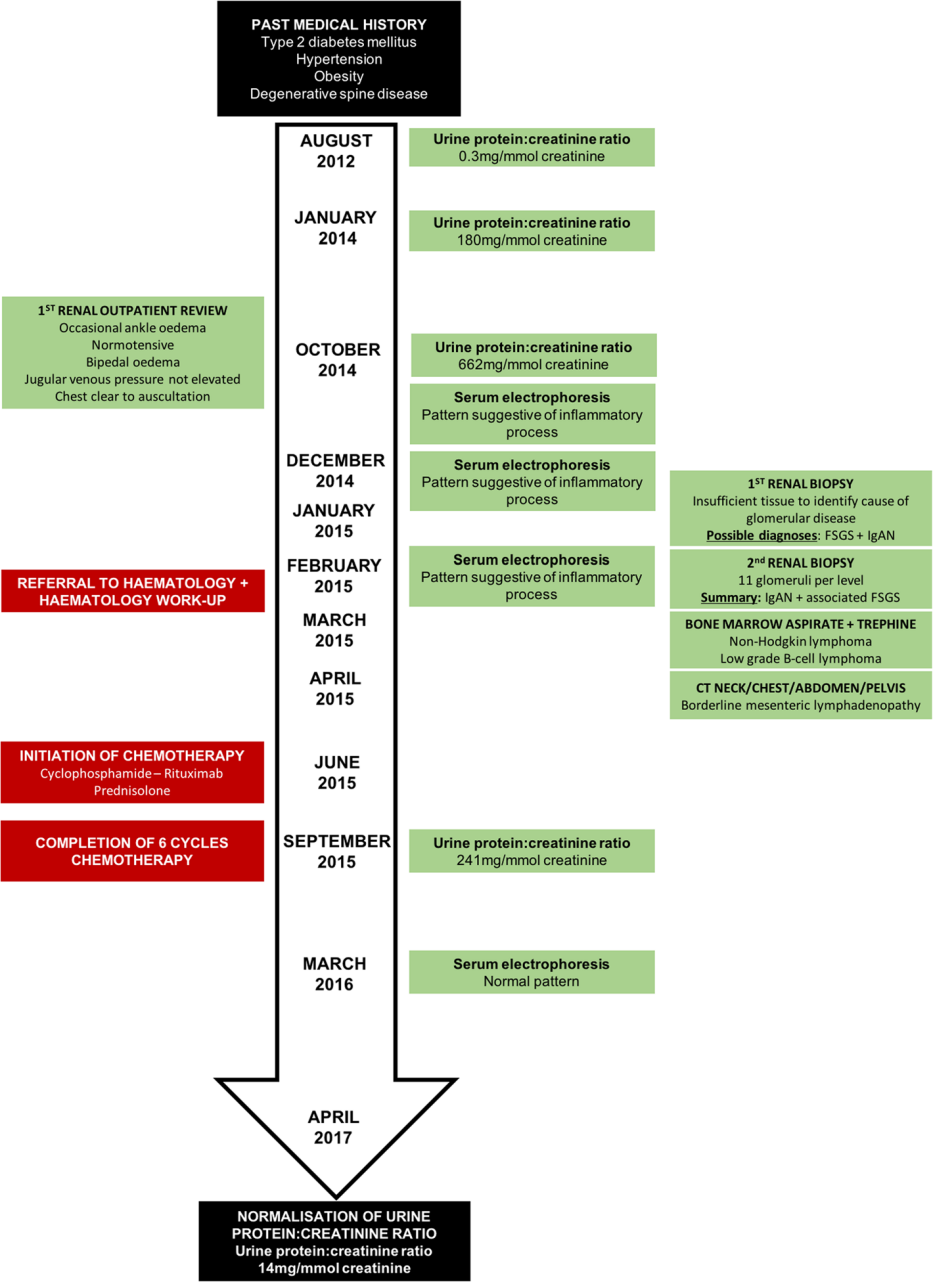
Timeline of clinical presentation, investigations and treatment. It is likely that the patient’s proteinuria initiated in 2013 and urine protein:creatinine ratio peaked at 662 mg/mmol prior to his first renal outpatient review in October, 2014. His second renal biopsy completed in February 2015 identified immunoglobulin A nephropathy (IgAN) and associated focal segmental glomerulosclerosis (FSGS). Bone marrow aspirate and trephine completed in March 2015 demonstrated low-grade B-cell lymphoma as the cause of his persistent abnormal serum electrophoresis pattern. Chemotherapy led to improvement in both serum electrophoresis pattern and urine protein excretion

Bone marrow biopsy showed normocellular marrow with mild lymphocytosis but no morphological evidence of plasma cell myeloma. Flow cytometry identified a clonal B-cell population suggesting a low grade B-cell lymphoma. Staging computer tomography scans of his neck, chest, abdomen and pelvis found some borderline mesenteric lymphadenopathy (clinically stage 1).

**Fig. 2:**
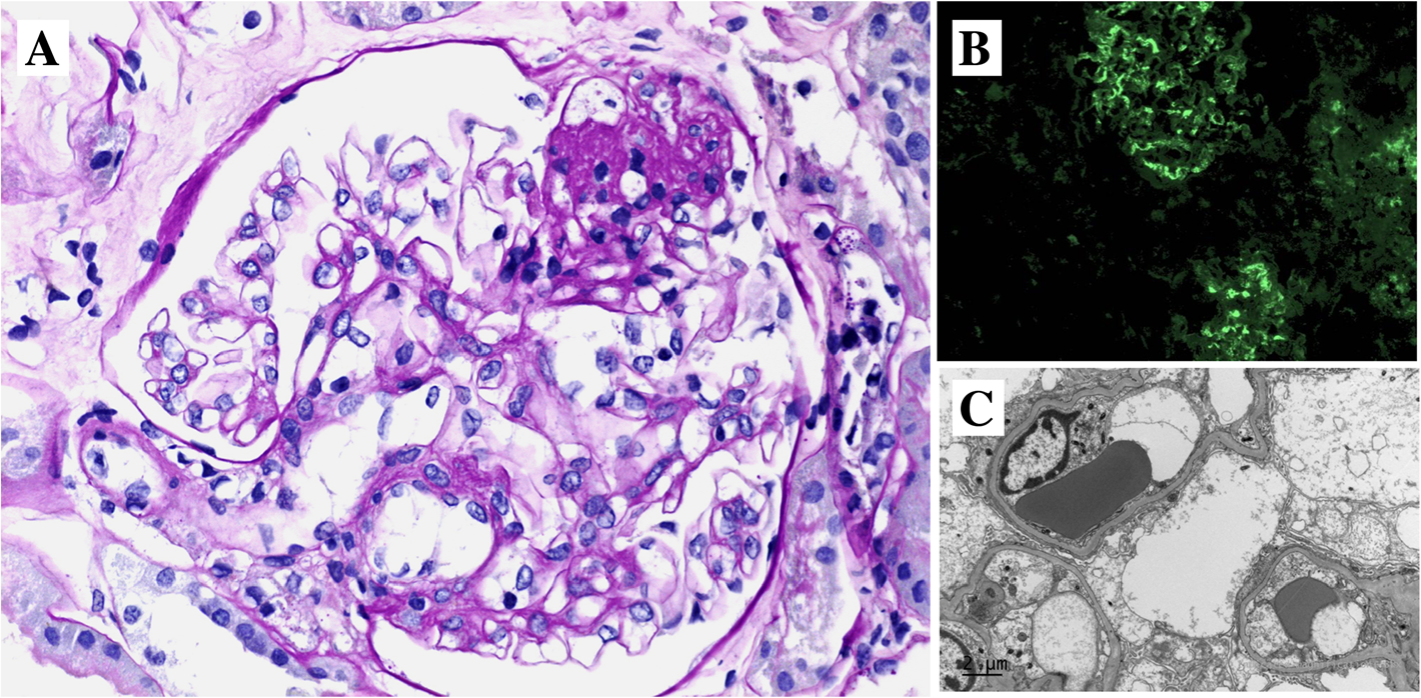
Kidney biopsy. **a** Light microscopy shows an area of segmental glomerular scarring with a foam cell and adhesion to Bowman’s capsule (upper right) away from the glomerular hilum. (Periodic acid–Schiff stain, 400× magnification). **b** Immunofluorescence shows mesangial reactivity of moderate intensity in two glomeruli. (200× magnification). **c** This electron microscopic image shows diffuse epithelial foot effacement over the basement membrane of three glomerular capillary loops. There is also podocytic swelling with focal microvillous transformation. (8,000× magnification)

The first renal biopsy demonstrated 4 glomeruli with minor mesangial hypercellularity but not expansion of the mesangial matrix. However, there was insufficient tissue to determine the cause of the patient’s glomerular disease. The second renal biopsy was performed a month later, yielding 11 glomeruli (Fig. 2). There was mild mesangial expansion but no mesangial hypercellularity, endocapillary proliferation nor crescent formation. One glomerulus was globally sclerosed. Three glomeruli showed areas of segmental sclerosis and adhesion to Bowman’s capsule. The lesions of segmental sclerosis were not perihilar in location. There was mild tubular atrophy involving 5% of the cortex and there was mild arteriolar hyalinosis. Immunofluorescence examination showed mesangial reactivity for IgA (moderate intensity), IgM (trace) and both kappa and lambda light chains (weak intensity). There was no reactivity for IgG, C3 or C1q. Electron microscopy showed extensive epithelial foot process effacement with microvillous change. A few small electron-dense deposits were found in the mesangium but not in glomerular capillary walls. Together, these findings indicated IgAN with associated FSGS.

In June 2015, the patient began 6 cycles of chemotherapy with cyclophosphamide (day 1: 750 mg/m^2^), rituximab (day 1: 375 mg/m^2^) and prednisolone (days 1–5: 50 mg/day). The patient did not demonstrate any side effects or complications from treatment. At the end of chemotherapy, the patient was in complete remission haematologically and urine protein creatinine ratio had decreased substantially to 241 mg/mmol creatinine. Over the next 14 months, his urine protein creatinine ratio continued to fall. His serum creatinine was 104 μmol/L, serum albumin was 38 g/L and urine protein excretion was 14 mg/mmol at his most recent follow-up, 26 months post-chemotherapy.

## Discussion and conclusions

Paraneoplastic glomerulonephritis in haematological malignancies is rare and tends to manifest as minimal change disease, membranous glomerulonephritis or membranoproliferative glomerulonephritis [12]. IgAN has previously been associated with T cell lymphoma [11, 13, 14], Hodgkin lymphoma [9, 15, 16], B-cell lymphoma [10, 17], leukaemia [10, 18] and multiple myeloma [19] in various case reports (Table 1).

**Table 1:** Summary of IgAN cases in haematological malignancy patients

Paper	Gender	Age (years)	Location	IgAN timing relative to malignancy Sx	Malignancy	Stage	SCr	Proteinuria	Haematuria	Serum IgA level	Kidney biopsy	Malignancy treatment	Renal recovery post malignancy treatment
Motoyama 2008 [10]	F	5	JPN	5 years post	ALL (undifferentiated)	NA	Normal	Present	Macro	Elevated	Crescents, mesangial proliferation, mesangial IgA deposits	Prednisolone, vincristine, methotrexate, 6-MP	No – recurrent microhaematuria w/o persistent proteinuria
Iwata 2006 [18]	M	28	JPN	8 years prior	ALL (undifferentiated)	NA	53 μmol/L	3 g/day	Micro	Normal	Glomerulosclerosis, mild mesangial proliferation, mesangial IgA deposits	Prednisolone, vincristine, daunorubicine, etoposide, mitoxantrone, cytarabine, methotrexate, peripheral stem cell transplantation	Yes – proteinuria decreased to 0.5 g/day at 16 months post CTx
Bergmann 2005 [9]	F	60	DEU	4 weeks post	Hodgkin lymphoma (mixed cellularity)	IIIA	681 μmol/L	2.3 g/day	Micro	Elevated	Crescents, diffuse mesangial proliferation, mesangial IgA deposits	Prednisolone, cyclophosphamide, etoposide, bleomycin, doxorubicin, vincristine, procarbazine	Yes – SCr, proteinuria within normal range
Khositseth 2007 [16]	M	14	THA	6 months post	Hodgkin lymphoma (nodular sclerosing)	IIIE	80 μmol/L	0.8 g/day	Micro	NR	Glomerulosclerosis, diffuse mesangial proliferation, mesangial IgA deposits	Cyclophosphamide, vincristine, doxorubicin, prednisolone	Yes – proteinuria normalized, urine RBC < 50/HPF
Cherubini 2001 [15]	M	44	ITA	1 year post	Hodgkin lymphoma (nodular sclerosing)	IV	354 μmol/L	4 g/day	Micro	Elevated	Crescents, mesangial IgA deposits	Prednisolone, nitrogen mustards, oncovin, procarbazine, adriamycin, bleomycin, vinblastine, dacarbazine	Yes – SCr within normal limits
Harada 2017 [11]	M	79	JPN	Same	Angioimmunoblastic T-cell lymphoma	IV	159 μmol/L	1.5 g/day	Micro	Elevated	Mesangial IgA deposits, lymphoma invasion	Prednisolone, cyclophosphamide, pirarubicine, vincristine	Yes – SCr, proteinuria, haematuria within normal range
Moe 1993 [13]	F	66	USA	6 months post	Mycosis fungoides	IV	High	1.0 g/day	Macro	NR	Crescents, diffuse mesangial proliferation, mesangial IgA deposits	Psoralen-ultraviolet A, allopurinol	No-IgAN presented after failed treatment
Ramirez 1981 [14]	M	70	USA	1 year post	Mycosis fungoides	IV	177 μmol/L	NR	Micro	NR	Glomerulosclerosis, mesangial proliferation, mesangial IgA deposits	NR	NR
Ramirez 1981 [14]	M	56	USA	3 years post	Mycosis fungoides	IV	Normal	NR	Micro	NR	Focal mesangial proliferation, mesangial IgA deposits	NR	NR
Motoyama 2008 [10]	M	12	JPN	3 years prior	Diffuse medium-sized B cell lymphoma	II	Normal	NR	Micro	Normal	Mild mesangial proliferation, mesangial IgA deposits	Prednisolone, cyclophosphamide, epirubicine, methotrexate, etoposide, cytarabine	No-recurrent macrohaematuria w/o persistent proteinuria
Mak 1998 [17]	M	62	HKG	Same	MALT B-cell lymphoma	IV	309 μmol/L	4.1 g/day	Macro	Elevated	Glomerulosclerosis, diffuse mesangial proliferation, mesangial IgA deposits, lymphoma invasion	Chlorambucil	Yes – SCr, proteinuria, haematuria within normal limits
Forslund 2007 [19]	M	76	FIN	5 years post	Multiple myeloma	III	752 μmol/L	4.0 g/day	Micro	Low	Mesangial proliferation, mesangial IgA deposits	Prednisolone, vincristine, cyclophosphamide, melphalan, adriamycin, dexamethasone, thalidomide, bortezomib	Yes – SCr 168 μmol/L 1 month post-treatment

*Abbreviations: 6-MP* 6-mercaptopurine, *ALL* acute lymphoblastic leukaemia, *CTx* chemotherapy, *DEU* Germany, *dx* diagnosis, *F* female, *FIN* Finland, *HKG* Hong Kong, *HPF* high power field, *IgA* immunoglobulin A, *IgAN* immunoglobulin A nephropathy, *JPN* Japan, *M* male, *Micro* microhaematuria, *Macro* macrohaematuria, *NA* not applicable, *NR* not reported, *RBC* red blood cells, *SCr* serum creatinine, *Sx* symptoms, *THA* Thailand, *USA* United States of America, *w/o* without

Staging methods: Hodgkin lymphoma: Ann Arbor staging system, Non-Hodgkin lymphoma: Ann Arbor staging system, Mycosis fungoides: Tumour Node Metastasis system, Multiple myeloma: Durie-Salmon system

The timing of IgAN development and subsequent remission post chemotherapy suggests that IgAN is a paraneoplastic phenomenon in these cases [9, 11, 15–19]. Current models of IgAN pathogenesis involve the production of anomalous galactosylated-deficient IgA1 and antibodies against the under-galactosylated IgA1 [20]. These components combine to form immune complexes in circulation which become deposited in the mesangium and cause complement activation, inflammation and mesangial cell proliferation. Haematological malignancies may contribute to IgAN pathogenesis via the production of abnormal IgA1 and/or associated autoantibodies. This is supported by clinical reports whereby 6 out of 12 haematological malignancy-associated IgAN patients exhibit elevated IgA levels. Furthermore, murine IgAN models link T helper 2 cell (T_H_2) dysfunction and cytokine accumulation with dysregulated IgA production [21]. Hodgkin lymphoma is associated with T cell expansion polarised towards a T_H_2-like phenotype and Reed-Sternberg cells constitutively express interleukin 13, a T_H_2 cytokine [22, 23]. Additionally, imbalances in T regulatory cells and their cytokines have been observed in IgAN patients potentially contributing to the pathogenesis of paraneoplastic IgAN in other T-cell lymphomas [24]. B cell lymphomas (as in this case) may participate in IgAN pathogenesis via the direct elaboration of anomalous IgA1 and autoantibodies via dysregulated clonal B-cell populations [25].

Here, we observed that paraneoplastic IgAN was associated with FSGS – a feature which distinguishes this case from previous reports. It is posited that immune complex-stimulated mesangial cells release mediators which incite podocyte injury thereby, contributing to segmental glomerulosclerosis in IgAN [26]. Notably, glomerular capsular adhesions, considered one of the first steps towards FSGS, is observed in up to 41% of primary IgAN renal biopsies [27]. The appearance of FSGS in primary and secondary IgAN suggests that podocyte injury likely occurs after the initial insult in IgAN pathogenesis [28]. While the patient’s FSGS could be attributed to type 2 diabetes mellitus, this is less likely as the patient had normal glycosylated haemoglobin levels for years prior to the onset of proteinuria. The biopsy also did not show features of diabetic nephropathy such as glomerular basement membrane thickening or mesangial expansion. Obesity-related FSGS is less likely considering that substantial improvements in proteinuria were observed post-chemotherapy in the absence of weight loss. In addition, the typical features of obesity-related FSGS, such as glomerulomegaly and perihilar sclerosis, were absent. Notably, IgAN with superimposed FSGS is associated with worse renal survival at 80 months compared to IgAN only patients (32.6% with FSGS versus 95.1% without FSGS) [29]. This could potentially explain the persistent mild proteinuria post-chemotherapy compared to reported cases.

Chemotherapy did not lead to renal improvement in 2 cases, suggesting that IgAN and malignancy can also be co-incidental [10]. In the first case, IgAN was diagnosed 5 years after the onset of acute lymphoblastic leukaemia and 4 months prior to the end of treatment, at which time the patient was already in remission. In the second case, IgAN was diagnosed and treated with dipyridamole 3 years prior to the onset of diffuse medium-sized B cell lymphoma. Considering the prolonged separation between IgAN development and haematological malignancy diagnosis, it is possible that the two pathologies are co-incidental. This raises the question: how does one differentiate between concurrent IgAN and paraneoplastic IgAN? Similar to idiopathic IgAN, lymphoma-associated IgAN predominantly presents with active urinary sediment (Table 1) and may rarely present with isolated nephrotic-range proteinuria (this case, [30, 31]). Notably, haematological malignancy chemotherapy and immunosuppressive IgAN treatment share many common features – treating one condition can improve the other even if the two conditions are co-incidental.

Previous cases have associated IgAN with late stage haematological malignancies suggesting that a threshold tumour load is required for the development of paraneoplastic IgAN. Here, the patient presented with IgAN features in the setting of asymptomatic low volume B-cell lymphoma. Combined with previous reports, this case demonstrates that paraneoplastic IgAN can occur with a broad range of haematological malignancies regardless of stage. This case illustrates the importance of meticulous haematological system work-up for patients presenting with IgAN. Recognition of paraneoplastic IgAN and early diagnosis of associated malignancy can be life-saving.

## Abbreviations

FSGS: Focal segmental glomerulosclerosis
Ig: Immunoglobulin
IgAN: Immunoglobulin A nephropathy
T_H_2: T helper 2 cell

## References

[1] Schena FP (1990). A retrospective analysis of the natural history of primary IgA nephropathy worldwide. Am J Med.

[2] Roberts IS (2014). Pathology of IgA nephropathy. Nat Rev Nephrol.

[3] da Silva LS, Almeida BL, de Melo AK, de Brito DC, Braz AS, Freire EA (2016). IgA nephropathy in systemic lupus erythematosus patients: case report and literature review. Rev Bras Reumatol Engl Ed.

[4] Sakellariou GT, Vounotrypidis P, Berberidis C (2007). Infliximab treatment in two patients with psoriatic arthritis and secondary IgA nephropathy. Clin Rheumatol.

[5] Forshaw MJ, Guirguis O, Hennigan TW (2005). IgA nephropathy in association with Crohn's disease. Int J Color Dis.

[6] Eykyn SJ, Jones NF, Nunan TO (1984). Brucellosis with mesangial IgA nephropathy: successful treatment with doxycycline and rifampicin. Br Med J (Clin Res Ed).

[7] Han SH, Kang EW, Kie JH, Yoo TH, Choi KH, Han DS, Kang SW (2010). Spontaneous remission of IgA nephropathy associated with resolution of hepatitis a. Am J Kidney Dis.

[8] Singh P, Khaira A, Sharma A, Dinda AK, Tiwari SC (2009). IgA nephropathy associated with pleuropulmonary tuberculosis. Singap Med J.

[9] Bergmann J, Buchheidt D, Waldherr R, Maywald O, van der Woude FJ, Hehlmann R, Braun C (2005). IgA nephropathy and hodgkin's disease: a rare coincidence. Case report and literature review. Am J Kidney Dis.

[10] Motoyama O, Kojima Y, Ohara A, Tsukimoto I, Ishikawa Y, Iitaka K (2008). IgA nephropathy associated with leukemia and lymphoma: report of two cases. Clin Exp Nephrol.

[11] Harada Y, Sakai K, Asaka S, Nakayama K (2017). Angioimmunoblastic T-cell lymphoma associated with IgA nephropathy. Intern Med.

[12] Lien YH, Lai LW (2011). Pathogenesis, diagnosis and management of paraneoplastic glomerulonephritis. Nat Rev Nephrol.

[13] Moe SM, Baron JM, Coventry S, Dolan C, Umans JG (1993). Glomerular disease and urinary Sezary cells in cutaneous T-cell lymphomas. Am J Kidney Dis.

[14] Ramirez G, Stinson JB, Zawada ET, Moatamed F (1981). IgA nephritis associated with mycosis fungoides. Report of two cases. Arch Intern Med.

[15] Cherubini C, Barbera G, Di Giulio SD, Muda AO, Faraggiana T (2001). Lymphomas and IgA nephropathy. Nephrol Dial Transplant.

[16] Khositseth S, Kanitsap N, Warnnissorn N, Thongboonkerd V (2007). IgA nephropathy associated with Hodgkin's disease in children: a case report, literature review and urinary proteome analysis. Pediatr Nephrol.

[17] Mak SK, Wong PN, Lo KY, Wong AK (1998). Successful treatment of IgA nephropathy in association with low-grade B-cell lymphoma of the mucosa-associated lymphoid tissue type. Am J Kidney Dis.

[18] Iwata Y, Wada T, Uchiyama A, Miwa A, Nakaya I, Tohyama T, Yamada Y, Kurokawa T, Yoshida T, Ohta S (2006). Remission of IgA nephropathy after allogeneic peripheral blood stem cell transplantation followed by immunosuppression for acute lymphocytic leukemia. Intern Med.

[19] Forslund T, Sikio A, Anttinen J (2007). IgA nephropathy in a patient with IgG lambda light-chain plasmacytoma: a rare coincidence. Nephrol Dial Transplant.

[20] Fabiano RC, Pinheiro SV, Simões E Silva AC. Immunoglobulin A nephropathy: a pathophysiology view. Inflamm Res. 2016;65(10):757–70. 10.1007/s00011-016-0962-x 27351940

[21] Inoshita H, Kim BG, Yamashita M, Choi SH, Tomino Y, Letterio JJ, Emancipator SN (2013). Disruption of Smad4 expression in T cells leads to IgA nephropathy-like manifestations. PLoS One.

[22] Kuppers R (2009). The biology of Hodgkin's lymphoma. Nat Rev Cancer.

[23] Ohshima K, Akaiwa M, Umeshita R, Suzumiya J, Izuhara K, Kikuchi M (2001). Interleukin-13 and interleukin-13 receptor in Hodgkin's disease: possible autocrine mechanism and involvement in fibrosis. Histopathology.

[24] Lin FJ, Jiang GR, Shan JP, Zhu C, Zou J, Wu XR (2012). Imbalance of regulatory T cells to Th17 cells in IgA nephropathy. Scand J Clin Lab Invest.

[25] Yuling H, Ruijing X, Xiang J, Yanping J, Lang C, Li L, Dingping Y, Xinti T, Jingyi L, Zhiqing T (2008). CD19+CD5+ B cells in primary IgA nephropathy. J Am Soc Nephrol.

[26] Cook HT (2011). Focal segmental glomerulosclerosis in IgA nephropathy: a result of primary podocyte injury?. Kidney Int.

[27] Hill GS, Karoui KE, Karras A, Mandet C, Duong Van Huyen JP, Nochy D, Bruneval P (2011). Focal segmental glomerulosclerosis plays a major role in the progression of IgA nephropathy. I. Immunohistochemical studies. Kidney Int.

[28] Fukuda A, Sato Y, Iwakiri T, Komatsu H, Kikuchi M, Kitamura K, Wiggins RC, Fujimoto S (2015). Urine podocyte mRNAs mark disease activity in IgA nephropathy. Nephrol Dial Transplant.

[29] El Karoui K, Hill GS, Karras A, Moulonguet L, Caudwell V, Loupy A, Bruneval P, Jacquot C, Nochy D (2011). Focal segmental glomerulosclerosis plays a major role in the progression of IgA nephropathy. II. Light microscopic and clinical studies. Kidney Int.

[30] Kim SM, Moon KC, Oh KH, Joo KW, Kim YS, Ahn C, Han JS, Kim S (2009). Clinicopathologic characteristics of IgA nephropathy with steroid-responsive nephrotic syndrome. J Korean Med Sci.

[31] Kim JK, Kim JH, Lee SC, Kang EW, Chang TI, Moon SJ, Yoon SY, Yoo TH, Kang SW, Choi KH (2012). Clinical features and outcomes of IgA nephropathy with nephrotic syndrome. Clin J Am Soc Nephrol.

